# Prevalence of Low Bone Density and Fracture Risk Assessed with the FRAX Tool in German Patients with Axial Spondyloarthritis: A Cross-Sectional Study

**DOI:** 10.3390/life16030439

**Published:** 2026-03-09

**Authors:** Elena Bischoff, Philipp Sewerin, Björn Bühring, Nikola Kirilov, Xenofon Baraliakos

**Affiliations:** 1 Rheumazentrum Ruhrgebiet Herne, Ruhr-Universität Bochum, 44649 Herne, Germany; 2 Department of Health Care, Trakia University, 6000 Stara Zagora, Bulgaria; 3 Bergisches Rheuma-Zentrum, Cellitinnen Krankenhaus St. Josef, 42105 Wuppertal, Germany; 4 Institute of Medical Informatics, Heidelberg University Hospital, 69120 Heidelberg, Germany; kirilov_9@web.de

**Keywords:** axSpA, BMD, FRAX

## Abstract

Introduction: Chronic inflammation in axial spondyloarthritis (axSpA) promotes osteoclast formation and bone resorption, leading to osteoporosis and an increased risk of fragility fractures. Osteoporotic fractures significantly impact the quality of life in patients with axSpA. While the Fracture Risk Assessment Tool (FRAX) is widely used to evaluate fracture risk, data on FRAX-based fracture risk assessment in axSpA, particularly in German patients, are limited. Objective: The primary objective of this study was to assess the prevalence of low bone mineral density (BMD) and fracture risk using FRAX for major osteoporotic fractures (MOF) and hip fractures (HF) in German patients with axSpA. Secondary objectives were to compare FRAX scores and BMD between genders and between patients with and without previous fractures, and to identify which FRAX parameters were most frequently abnormal. Materials and Methods: This retrospective study analyzed demographic and clinical data, along with DXA-measured BMD, T-scores and Z-scores of the lumbar spine and femoral neck in 58 axSpA patients aged 43–81 years from routine clinical practice. Calculations for MOF and HF were performed using the FRAX model for Germany. Low BMD was defined as a T-score < −1 SD or a Z-score < −2 SD. Statistical analyses included independent *t*-tests and chi-square tests. Results: The mean age of patients was 65 years with a mean BMI of 29.5 kg/m^2^. The prevalence of low BMD was 44.8% at the lumbar spine and 60.4% at the femoral neck. Overall, 10 (17.2%) patients reported previous fractures of the spine, forearm, hip, or shoulder. Female patients had higher FRAX scores for MOF (8.2%) than males (6.8%, *p* = 0.02), while male patients had higher FRAX scores for HF (2.8% vs. 2%, *p* = 0.04). There was no significant difference in BMD between patients with or without a history of fracture. However, patients with previous fractures had significantly higher FRAX scores for MOF (10.2%) compared to those without fractures (7.3%, *p* = 0.030); the difference in HF scores was not statistically significant (3.5% vs. 2%, *p* = 0.056). Conclusions: This study highlights the elevated fracture risk in axSpA patients assessed with FRAX. In this cohort, BMD alone was not associated with fracture history, suggesting that other factors—such as age, sex, glucocorticoid exposure, and prior fractures—may play a more prominent role. FRAX provides a valuable tool for evaluating fracture risk in axSpA, emphasizing the importance of a comprehensive assessment that incorporates both clinical risk factors and BMD.

## 1. Introduction

The Fracture Risk Assessment Tool (FRAX) is the most widely used calculator for estimating the 10-year probability of major osteoporotic fractures (MOF) and hip fractures (HF) globally. It was developed as a country-specific risk estimator for more than 65 countries [1,2,3]. Intervention thresholds of FRAX have been published to guide physicians in determining whether patients require treatment for osteoporosis. FRAX can be calculated with or without inclusion of bone mineral density (BMD), and both approaches predict future fracture risk [4,5]. Previous studies in patients with axial spondyloarthritis (axSpA) have applied FRAX, including BMD [6,7,8,9,10]. In Germany, FRAX is less commonly used because the German osteoporosis guideline published by the Dachverband Osteologie (DVO) employs a different method to calculate fracture risk. Consequently, only limited FRAX-based data are available for German axSpA patients. Accurate fracture risk assessment is particularly important in axSpA, as disease-specific mechanisms substantially contribute to bone fragility.

Chronic inflammation contributes to bone loss in axSpA through the secretion of pro-inflammatory cytokines and receptor activator of nuclear factor κB ligand (RANKL), which enhances osteoclast formation. The resulting increase in bone resorption plays a central role in the pathogenesis of osteoporosis and subsequent fractures. Osteoporosis is defined as a T-score < −2.5 standard deviations (SD) compared to healthy young adults according to the World Health Organization and is a common complication of inflammatory rheumatic diseases [11,12,13]. Osteoporotic fractures significantly impair quality of life due to disability and are associated with increased morbidity and mortality [14]. Previous studies have shown that patients with axSpA have an increased risk of osteoporotic fractures due to disease activity, rigidity of the axial skeleton, and frequent falls resulting from impaired balance [15,16]. The prevalence of vertebral fractures in patients with axSpA varies widely from 1.4% to 58%, depending on selection criteria. Moderate to severe vertebral fractures occur in more than 10% of patients younger than 40 years with a disease duration of 5 years [17]. In a cohort of 5909 patients with radiographic axSpA (r-axSpA) and 28,671 matched controls, patients with r-axSpA showed a significantly higher incidence and earlier development of hip fractures than matched cohorts [18].

The primary objective of this study was to assess the prevalence of low BMD and fracture risk using FRAX for MOF and HF, including BMD, in a German axSpA cohort. Secondary objectives were to compare FRAX risk and BMD between genders and between individuals with and without fractures, and to identify which parameters included in FRAX were most commonly abnormal and could therefore be considered fracture risk factors in axSpA patients.

## 2. Materials and Methods

This retrospective study was conducted to evaluate demographic and clinical data, along with DXA-measured BMD, T-scores, and Z-scores of the lumbar spine and femoral neck of patients diagnosed with axSpA who were admitted to a large tertiary university rheumatology hospital (Rheumazentrum Ruhrgebiet, Ruhr-University Bochum, Germany) between 2022 and 2024. Patients were mainly admitted to the hospital for clinical reasons such as pain and increased disease activity, requiring treatment adaptation of any kind, such as escalation from NSAIDs to a biologic (b-) or targeted synthetic (ts-) disease-modifying anti-rheumatic drug (DMARD) or a switch of a previously failed b- or ts-DMARD.

Criteria for inclusion in the analysis were confirmed diagnosis of axial SpA, age between 40 and 90 years, as well as availability of a dual-energy X-ray absorptiometry (DXA) scan of the lumbar spine and femoral neck. DXA examinations were performed as part of the clinical routine, where indicated, at the discretion of the treating rheumatology consultant at the time of the clinical examination.

### 2.1. Data Collection

Demographic and anthropometric data, including age, sex, and body mass index (BMI), were collected for all patients. BMD measurements at the lumbar spine and left femoral neck were performed using a DXA scan, General Electric Lunar Prodigy (GE Healthcare, Madison, WI, USA). The following parameters were derived from the DXA measurements:

- T-scores: Standard deviations relative to a young, healthy reference population.
- Z-scores: Standard deviations relative to an age-matched reference population.

Low BMD was defined as a T-score < −1 standard deviation (SD) or a Z-Score of <−2 SD.

In patients with axial spondyloarthritis (axSpA), structural spinal changes, including syndesmophytes and other osteoproliferative alterations, may lead to artificially increased lumbar spine BMD values when assessed by DXA. Consequently, lumbar spine DXA measurements may not fully reflect the underlying bone fragility in this patient population. This methodological limitation of spinal DXA was considered when interpreting the results. In addition, the potential role of the Trabecular Bone Score (TBS) was acknowledged as a complementary parameter for assessing bone quality beyond BMD, as TBS provides indirect information on trabecular microarchitecture.

### 2.2. Fracture Risk Assessment

The Fracture Risk Assessment Tool (FRAX) was used to calculate the 10-year probability of major osteoporotic fractures (MOF) and hip fractures (HF) for each patient. The history of previous fractures was documented through a review of medical records and morphometric vertebral fracture assessments (VFA) from DXA scans. Other risk factors, including a parental history of hip fracture, current smoking, alcohol consumption of three or more units per day, current or past corticosteroid use (for more than three months at a daily prednisolone dose of 5 mg or higher), rheumatoid arthritis, and secondary osteoporosis, were marked as “yes” if documented in the medical records; otherwise, they were marked as “no”. FRAXplus calculator (https://www.fraxplus.org/calculation-tool), accessed on 2 January 2025, was used to evaluate the 10-year fracture risk for MOF and for HF. The FRAX calculations were performed using the FRAX model available for Germany. Patients were further classified into two groups based on whether they had a history of previous fractures.

### 2.3. FRAX-Based Intervention Thresholds

We applied the fixed probability approach for FRAX-based intervention thresholds, which is used in the USA and Canada, and considers both sexes regardless of age. This method sets intervention thresholds at a 20% FRAX 10-year probability for MOF and 3% for HF. The Bone Health and Osteoporosis Foundation (BHOF) recommends treatment for patients with hip or vertebral fractures, osteoporosis (T-score ≤ −2.5 at the femoral neck, total hip, or lumbar spine), or osteopenia with a 10-year fracture risk ≥ 20% for MOF or ≥3% for HF in postmenopausal women and men over 50 years [2].

In contrast, the German DVO defines different thresholds for the “DVO-equivalent risk level” when using FRAX with BMD. One study among male patients identified thresholds of ≥6.7% for MOF and ≥2.1% for HF, while another study in female patients reported thresholds of ≥10% for MOF and ≥2.6% for HF [19,20]. To explore these differences, we assessed the distribution of patients across different FRAX threshold groups, categorizing MOF risk into <10%, 10–20%, and >20%, and HF risk into <2.6%, 2.6–3%, and >3%.

#### Sex-Specific Analysis

Clinical and bone health parameters, including BMI, BMD, T-scores, Z-scores, presence of previous fractures, and FRAX scores, were analyzed separately for male and female patients due to well-known gender differences in osteoporosis.

### 2.4. Fracture-Status Analysis

Previous fractures were defined as a history of any of the following: spine, forearm, hip, or shoulder fractures. A morphometric vertebral fracture, identified through radiographic imaging, was also classified as a previous fracture.

#### Statistical Analysis

Data were analyzed to evaluate differences in means, standard deviations (SD), minimum and maximum values, standard error of mean, as well as proportions and associations between variables. Independent *t*-tests and chi-square tests were used in the statistical analysis. A *p*-value < 0.05 was considered statistically significant. Statistical analyses were performed using SPSS software (version 23, IBM Corp., Armonk, NY, USA).

## 3. Results

The mean age of all 58 patients was 65 years ± 9 years, range (43–81 years), mean BMI was 29.5 kg/m^2^ ± 6.7 kg/m^2^ (range 16.3–46.5 kg/m^2^). The mean BMD of the total lumbar spine was 1.111 ± 0.229 g/cm^2^, range (0.610–1.695 g/cm^2^). The mean T-score of the total lumbar spine was −0.7 ± 0.2 standard deviations (SD), range (−4.7 SD and 4 SD). The mean Z-score of the spine was 0 SD ± 0.2, range (−3.7 SD to 4.7). The mean BMD, T-score, and Z-score of the left femoral neck were as follows: mean BMD = 0.550 g/cm^2^ ± 0.158 g/cm^2^, mean T-score = −1.3 SD ± 1.2 SD, mean Z-score = −0.4 SD ± 1.2 SD. Patients had a mean FRAX for MOF of 7.8% ± 1.2% and FRAX for HF of 2.3% ± 2.2%, Table 1.

**Figure 1:**
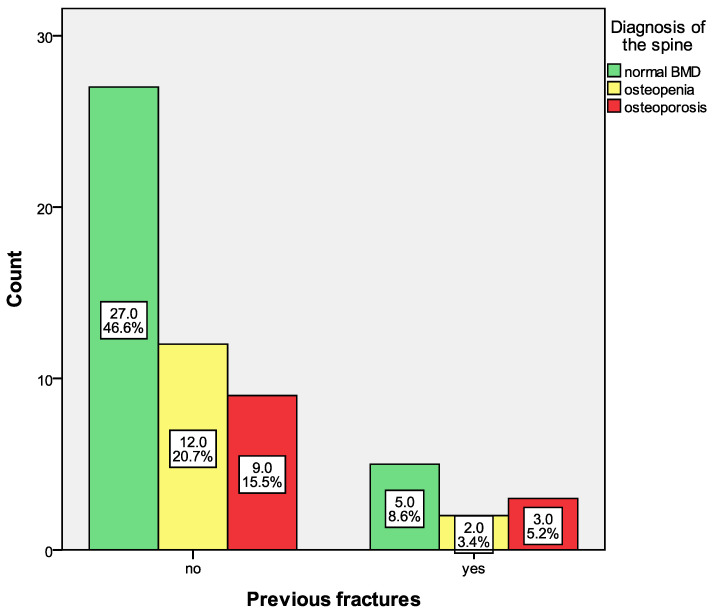
Prevalence of osteoporosis of the spine in patients without and with previous fractures.

Forty (68.97%) were female. Ten patients (17.2% of all 58 patients) had reported previous fractures; 5 were female (12.5% of all female patients), and 5 were male (27.8% of all male patients), see Figure 1. Male patients were older (mean age of 67 years) than female patients (mean age of 64 years). The mean BMI of male patients was numerically lower (28.9 kg/m^2^) than that of female patients (29.7 kg/m^2^); however, this difference did not reach statistical significance. The BMD, T-score, and Z-score of the lumbar spine were lower (BMD = 1.063 kg/cm^2^, T-score = −1 SD, Z-score = −0.1 SD) in female patients compared to males (BMD = 1.226 kg/cm^2^, T-score = 0 SD, Z-score = 0.1 SD). A significant difference was observed only in the mean BMD values between male and female subjects (*p* = 0.037). Similarly, BMD, T-score, and Z-score of the left femoral neck (BMD = 0.818 kg/cm^2^, T-score = −1.4 SD, Z-score = −0.4 SD) were lower in females compared to males (BMD = 0.915 kg/cm^2^, T-score = −1.2 SD, Z-score = 0.3 SD), although these differences lacked statistical significance. The mean FRAX for MOF of the female patients was higher (8.2%) than that of male patients (6.8%), whereas the mean FRAX for HF (2.8%) of the male patients was higher than that of the female patients (2%), but these differences were not significant, see Table 1.

Overall, taking both lumbar spine and left femur neck BMD into account, osteopenia was present in 21 patients (36.2%) and osteoporosis in 15 patients (25.9%). Thirty-six patients (62.1%) had low BMD (T-score below −1.0).

The prevalence of osteopenia of the lumbar spine was 24.1% (14 of 58 patients), and that of osteoporosis was 20.7% (12 of 58 patients). The overall prevalence of low BMD of the spine was 44.8% (26 of 58 patients).

Patients with previous osteoporotic fractures did not show a higher prevalence of osteoporosis of the spine compared to those without previous fractures, *p* = 0.772. Fifty percent (5 of 10 patients) of patients with fractures showed normal BMD of the spine, 20% osteopenia (2 of 10 patients), and 30% (3 of 10 patients) osteoporosis, Figure 1.

The prevalence of osteopenia of the left femoral neck was 41.4% (24 of 58 patients), and that of osteoporosis was 19% (11 of 58 patients). The overall prevalence of low BMD of the femoral neck was 60.4% (35 of 58 patients).

**Figure 2:**
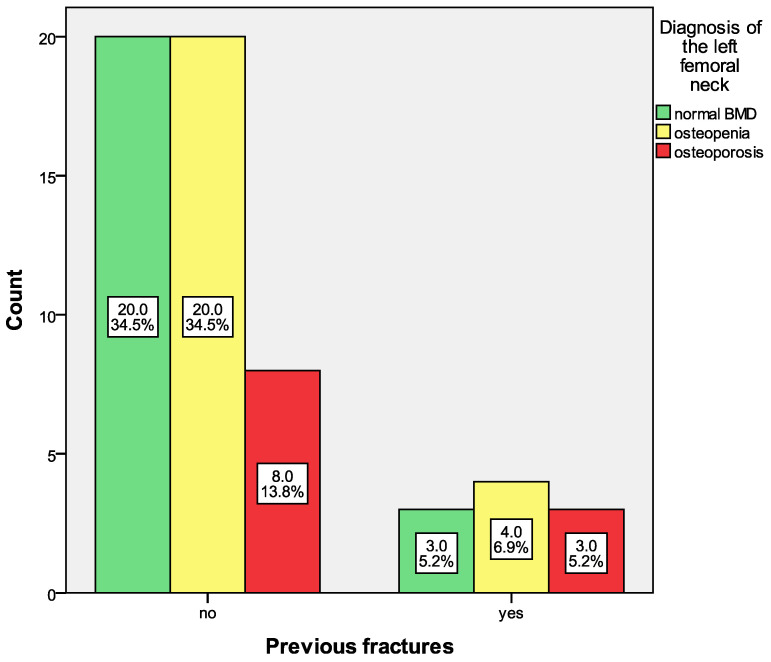
Prevalence of osteoporosis of the left femoral neck in patients without and with previous fractures.

Patients with previous fractures did not show a higher prevalence of osteoporosis of the left femoral neck compared to those without previous fractures, *p* = 0.587. According to the T-score of the femoral neck, the group of patients with fractures predominated those with osteopenia (40%) compared to normal BMD (30%) and osteoporosis (30%), Figure 2.

The mean age was significantly higher in patients with fractures (70 ± 8 years, range 55–81) compared to those without (64 ± 9 years; *p* = 0.046). No significant differences were observed in lumbar spine BMD (1.116 g/cm^2^ ± 0.214 vs. 1.087 g/cm^2^ ± 0.308) or T-scores (−0.6 ± 1.8 vs. −1.0 ± 2.4), *p* > 0.05.

Similarly, left femoral neck BMD (0.854 g/cm^2^ ± 0.157 vs. 0.829 g/cm^2^ ± 0.167) and T-scores (−1.2 ± 1.2 vs. −1.6 ± 1.1) did not differ significantly between groups with and without previous fractures, *p* > 0.05. The FRAX score for MOF was significantly higher in patients with fractures (10.2% ± 4.7) compared to those without (7.3% ± 3.6; *p* = 0.030). Although the FRAX score for hip fractures was higher in the group with fractures compared to those without fractures (3.5% ± 2.3 vs. 2.0% ± 2.1), this difference did not reach statistical significance (*p* = 0.056), Table 2.

When looking at the different parameters included in FRAX, we found that only age and FRAX for MOF differed significantly between the groups with and without fractures (*p* = 0.046 for age; *p* = 0.030 for FRAX MOF). All other parameters showed no significant differences.

### Risk Factors for FRAX and Treatment Thresholds

In our cohort, 15 patients (25.9%) were smokers, and 8 patients (13.8%) were treated with corticosteroids. No patients had a diagnosis of rheumatoid arthritis or secondary osteoporosis. Additionally, 3 patients (5.2%) reported alcohol consumption of three or more units per day.

FRAX scores for both MOF and HF differed significantly between patients with and without corticosteroid use. Patients on corticosteroids had a higher mean MOF risk of 9.4% compared to 6.3% in non-users (*p* = 0.04). Similarly, for HF, corticosteroid users had a mean risk of 3.1%, while non-users had a lower mean risk of 1.7% (*p* = 0.03). Smoking and alcohol consumption did not show any significant association with FRAX scores.

Regarding treatment thresholds, 43 patients (74.1%) had a FRAX MOF risk of <10%, while 15 patients (25.9%) had a risk between 10 and 20 patients had a FRAX MOF risk exceeding 20%. For FRAX HF, 40 patients (69%) had a risk of <2.6%, 2 patients (3.4%) fell within 2.6–3%, and 16 patients (27.6%) had a risk exceeding 3%.

## 4. Discussion

This study aimed to investigate the prevalence of low BMD in patients with axSpA and to assess the fracture risk in this patient population using FRAX with the inclusion of BMD. Furthermore, associations between calculated fracture risk and risk factors, including past fractures, were explored.

We report for the first time that the mean FRAX score for HF in our fractured patients was found to be within the treatment threshold, despite the result not reaching statistical significance. This finding highlights the potential role of clinical factors, including previous fractures, in fracture risk estimation and suggests a need for further investigation into the factors that might influence fracture risk beyond BMD alone.

The prevalence of low BMD in our study was lower than that reported by Ataş et al. [21] and comparable to that reported by Ramirez et al. [22]. In patients with axial spondyloarthritis (axSpA), the interpretation of lumbar spine bone mineral density (BMD) measured by dual-energy X-ray absorptiometry (DXA) requires careful consideration. Structural spinal changes characteristic of axSpA, such as syndesmophytes and other osteoproliferative alterations, may lead to artificially elevated lumbar spine BMD values. As a result, DXA measurements at the lumbar spine may underestimate the presence of reduced bone strength and fracture risk in this patient population.

In this context, complementary approaches that provide information beyond BMD may be of particular relevance. The TBS, which is derived from DXA images, has been proposed as an additional parameter reflecting trabecular microarchitecture and bone quality. Therefore, TBS may offer further insight into bone fragility in patients with axSpA and could improve the assessment of skeletal health in this population.

While 17.2% of the cohort in our study reported previous fractures, there was no significant association between previous fractures and the prevalence of osteoporosis or low BMD, neither at the lumbar spine nor at the femoral neck. This suggests that low BMD may not be directly associated with fractures in this cohort, as DXA measurements can be affected by osteoproliferative changes in the spine and hip in axSpA patients. These changes may limit the accuracy of BMD in predicting fracture risk, and alternative methods like Osteo-CT may offer a more reliable assessment. Factors beyond BMD (such as disease-related inflammation or medication use) are also contributing to fracture risk.

Our findings suggest that corticosteroid use is associated with an increased risk of both MOF and HF in axSpA patients. In contrast, smoking and alcohol consumption showed no significant association with FRAX scores. Similarly, Kim et al. did not identify smoking and alcohol consumption as significant risk factors for FRAX, whereas corticosteroid use remained a significant factor in their multivariate analysis [23]. There is a lack of studies investigating the association of these risk factors with fracture risk as assessed by FRAX. Most existing research focuses on disease-related inflammation and its impact on fracture risk. Previous studies in patients with inflammatory diseases have shown that elevated levels of proinflammatory factors and disease activity can independently influence bone metabolism and fracture risk, even in patients without manifested osteoporosis [24,25,26]. Future studies are needed to investigate how the disease activity, as well as risk factors included in FRAX in SpA patients, are associated with osteoporotic fractures in cases of normal BMD.

The overall mean FRAX score for MOF in our study was 7.8%, and that for HF was 2.3%, which falls within the normal range according to the defined FRAX treatment thresholds. Pharmacological intervention should be considered if a FRAX-calculated 10-year probability of HF is greater than 3% or a 10-year probability of a MOF is greater than 20% [14]. Mean FRAX for MOF and mean FRAX for HF in our study were higher than those reported by Henchiri et al. (0.36% for MOF and 0.3% for HF) [6]. A Swedish study including AS patients reported a higher mean FRAX for MOF (9.9%) compared to our study and a similar mean FRAX for HF (2.4%) [27]. These contradictory results may be explained by differences in the population characteristics and different fracture incidence in the different countries. However, all studies in SpA patients reported mean FRAX scores within the normal range based on FRAX treatment thresholds for the general population. A key consideration is that the average age in these cohorts tends to be lower than in many osteoporosis studies, where fracture risk is typically assessed in older populations. Since age is a major determinant of FRAX scores, a younger study population may inherently show lower fracture probabilities, even in the presence of other risk factors such as inflammation or corticosteroid use. This could lead to an underestimation of fracture risk in axSpA patients, particularly if additional disease-related factors contribute to bone fragility beyond what FRAX captures. This may also be explained by the findings of García et al., who reported that incorporating BMD into the FRAX calculation can influence fracture risk estimates in SpA patients [28].

Our data indicate that FRAX may underestimate fracture risk in patients with axSpA, as it does not account for chronic inflammation such as elevated CRP levels, which can independently affect bone metabolism and fracture susceptibility. This suggests that additional clinical “adjustment factors,” including measures of disease activity and inflammatory markers, may be necessary to more accurately assess fracture risk in axSpA beyond what is captured by FRAX alone. Incorporating such factors could improve risk stratification and help guide clinical decision-making for osteoporosis management in this population.

As expected, patients with fractures in our study had a significantly higher FRAX score for MOF, given that a previous fracture is a key risk factor in the FRAX algorithm. Interestingly, no significant difference was observed in hip fracture risk, which may be due to the relatively younger age of our cohort. Since hip fractures are strongly linked to age-related factors such as increased fall risk, the lack of a significant difference in hip fracture risk might reflect the relatively younger age of our cohort. This highlights the strong predictive ability of the FRAX tool for MOF. In contrast, the FRAX score for HF did not show a significant difference between fractured and non-fractured, although the results were approaching significance.

Comparing male and female patients, and taking into account that axSpA is a disease with strong male predominance, it was observed that female patients had lower lumbar spine and femoral neck BMD compared to male patients, which aligns with the well-established gender differences in bone density, with generally having lower BMD due to hormonal changes associated with menopause. Additionally, it has been suggested that women with axSpA exhibit fewer radiographic changes, such as syndesmophytes or spinal ankylosis, which can falsely elevate BMD values in DXA measurements. This may partly explain the observed differences in BMD between male and female patients. The higher FRAX score for MOF in female patients reflects this difference in risk, suggesting that while female patients with axSpA are at higher risk of MOF, male patients may still experience substantial risk, especially for HF. These results are consistent with the study by Tsur et al., which found that female patients with AS had a lower odds ratio for the association between AS and hip fractures (1.48) compared to male patients (1.65) [19].

The lack of a significant difference in BMD, T-scores, and Z-scores between fractured and non-fractured patients shows that factors such as age and corticosteroid use included in the FRAX calculator play a more important role than BMD alone for fracture prediction. These results are consistent with the findings of Iacovantuono et al., who reported higher FRAX scores when BMD was excluded from the calculation [29].

The fixed probability thresholds approach, widely used in the USA and Canada, provided a structured framework for assessing fracture risk in our cohort. This method defines intervention thresholds as a 20% 10-year probability for MOF and 3% for HF, irrespective of age or sex. Applying these criteria in our study, we were unable to identify any patients eligible for treatment based on FRAX MOF. However, when considering FRAX for HF, 16 patients (27.6%) exceeded the 3% risk threshold. In contrast, using the “DVO-equivalent risk level” and the adjusted thresholds applied when incorporating BMD into FRAX, we observed a different risk stratification. Specifically, 25.9% of the patients were classified as high risk for MOF, and 31% for HF.

### 4.1. Strengths

A major strength of our study is the well-defined objective to assess both the prevalence of low bone density and fracture risk using FRAX for MOF and FRAX for HF in patients with axSpA, which is an important and relatively underexplored area of research. The study gathers a wide range of clinical, demographic, and bone health data, including detailed information on age, sex, BMI, BMD, T-scores, Z-scores, and fracture history. This is important to build a comprehensive picture of each patient’s bone health and fracture risk.

### 4.2. Limitations

One potential limitation of the study is the relatively small sample size, particularly when stratifying patients by sex or fracture history, which might have limited the power to detect smaller differences in BMD between groups. Furthermore, although the FRAX model is a valuable tool for assessing fracture risk, it does not account for all factors, such as disease activity or medication history, that could influence fracture risk in patients with axSpA. The retrospective nature of the study may result in the omission of unreported risk factors or conditions. Another limitation is the disproportionate number of females compared to males in the cohort, which may not reflect a typical axSpA population. However, it seems that the clinical decision to measure osteoporosis was more frequently applied to females, which aligns with expectations and daily clinical practice for assessing osteoporosis risk. Due to the relatively small sample size, this study may have been underpowered to detect significant differences in BMD and T-scores between patients with and without fractures. Therefore, the absence of statistically significant differences should be interpreted with caution, as a type II error (failure to detect an existing association) cannot be excluded. The use of biologics, including TNF inhibitors and other bDMARDs, was not assessed in this study because these therapies are not included in the FRAX algorithm, as they can improve BMD by reducing systemic inflammation in axSpA. This represents a limitation when interpreting fracture risk in our cohort.

## 5. Conclusions

While BMD and fracture risk assessments in patients with axSpA indicate some degree of bone loss, they do not fully explain the fracture history in this cohort. The results highlight the importance of using comprehensive fracture risk tools like FRAX and considering clinical factors in managing the bone health of these patients. Future research should explore the complex interplay of these factors and the long-term fracture risk in this unique patient population.

## Figures and Tables

**Table 1:** Patients’ characteristics, DXA assessments of the lumbar spine and femoral neck, as well as fracture risk stratified by sex (female, male) and the overall cohort.

Characteristics		Mean	Minimum	Maximum	Standard Deviation	*p*-Value
Age (years)	Female	64	43	81	9	0.253
	Male	67	51	81	9	
	Total	65	43	81	9	
Weight (kg)	Female	77	51	120	19	0.005
	Male	91	50	115	15	
	Total	82	50	120	19	
Height (cm)	Female	162	143	175	6	<0.001
	Male	178	164	202	9	
	Total	167	143	202	10	
BMI (kg/m^2^)	Female	29.7	18.5	46.5	7.4	0.676
	Male	28.9	16.3	35.5	4.8	
	Total	29.5	16.3	46.5	6.7	
BMD of total lumbar spine (g/cm^2^)	Female	1.063	0.610	1.657	0.188	0.037
	Male	1.226	0.814	1.695	0.277	
	Total	1.111	0.610	1.695	0.229	
T-score of total lumbar spine (SD)	Female	−1.0	−4.7	4.0	1.6	0.059
	Male	0	−3.4	4.0	2.3	
	Total	−0.7	−4.7	4.0	1.9	
Z-score of total lumbar spine (SD)	Female	−0.1	−3.1	4.4	1.5	0.698
	Male	0.1	−3.7	4.7	2.3	
	Total	0	−3.7	4.7	1.8	
BMD of left femoral neck (g/cm^2^)	Female	0.818	0.619	1.115	0.113	0.084
	Male	0.915	0.594	1.320	0.212	
	Total	0.850	0.594	1.320	0.157	
T-score left femoral neck (SD)	Female	−1.4	−3.0	1.1	0.9	0.658
	Male	−1.2	−3.7	1.9	1.6	
	Total	−1.3	−3.7	1.9	1.2	
Z score of left femoral neck (SD)	Female	−0.4	−1.9	2.1	0.9	0.230
	Male	−0.3	−2.5	2.7	1.7	
	Total	−0.4	−2.5	2.7	1.2	
FRAX for MOF (%)	Female	8.2	2.9	17.2	4.3	0.197
	Male	6.8	1.8	12.1	3.0	
	Total	7.8	1.8	17.2	3.9	
FRAX for HF (%)	Female	2.0	0.1	7.3	2.1	0.258
	Male	2.8	0.04	8.6	2.5	
	Total	2.3	0.04	8.6	2.2	

**Table 2:** Characteristics of patients with and without fractures: mean, standard deviation (SD), standard error (SE), minimum, maximum, and *p*-value indicating the significance between the two groups for each variable.

Characteristics		Mean	Std. Deviation	Std. Error	Minimum	Maximum	*p*-Value
Age (years)	Without fractures	64	9	1.3	43	81	0.046
	With fractures	70	8	2.7	55	81	
	Total	65	9	1.2	43	81	
BMD of total lumbar spine (g/cm^2^)	Without fractures	1.116	0.214	0.030	0.814	1.695	NS
	With fractures	1.087	0.308	0.103	0.610	1.469	
	Total	1.111	0.229	0.030	0.610	1.695	
T-score of total lumbar spine (SD)	Without fractures	−0.6	1.8	0.3	−3.4	4.0	NS
	With fractures	−1.0	2.4	0.8	−4.7	2.0	
	Total	−0.7	1.9	0.3	−4.7	4.0	
Z-score of total lumbar spine (SD)	Without fractures	0	1.8	0.3	−3.7	4.7	NS
	With fractures	−0.2	1.9	0.6	−3.1	2.2	
	Total	0	1.8	0.2	−3.7	4.7	
BMD of left femoral neck (g/cm^2^)	Without fractures	0.854	0.157	0.023	0.594	1.320	NS
	With fractures	0.829	0.167	0.053	0.619	1.121	
	Total	0.849	0.158	0.021	0.594	1.320	
T-score left femoral neck (SD)	Without fractures	−1.2	1.2	0.2	−3.7	1.9	NS
	With fractures	−1.6	1.1	0.4	−3.0	0.4	
	Total	−1.3	1.2	0.2	−3.7	1.9	
Z score of left femoral neck (SD)	Without fractures	−0.3	1.2	0.2	−2.5	2.7	NS
	With fractures	−0.4	1.3	0.4	−1.9	1.9	
	Total	−0.4	1.2	0.2	−2.5	2.7	
FRAX for MOF (%)	Without fractures	7.3	3.6	0.5	1.84	16.9	0.030
	With fractures	10.2	4.7	1.5	4.8	17.2	
	Total	7.8	3.9	0.5	1.9	17.2	
FRAX for HF (%)	Without fractures	2.0	2.1	0.3	0.04	8.6	0.056
	With fractures	3.5	2.3	0.7	1.2	7.3	
	Total	2.3	2.2	0.3	0.04	8.6	
BMI (kg/m^2^)	Without fractures	29.8	7.3	1.0	16.3	46.5	NS
	With fractures	27.8	3.3	1.0	22.7	33.8	
	Total	29.5	6.7	0.9	16.3	46.5	

## Data Availability

The authors confirm that the data supporting the findings of this study are not publicly available due to privacy and ethical restrictions.
